# Evaluation of the online Beverage Frequency Questionnaire (BFQ)

**DOI:** 10.1186/s12937-018-0380-8

**Published:** 2018-08-01

**Authors:** Lana Vanderlee, Jessica L. Reid, Christine M. White, Erin P. Hobin, Rachel B. Acton, Amanda C. Jones, Meghan O’Neill, Sharon I. Kirkpatrick, David Hammond

**Affiliations:** 1 Department of Nutritional Sciences, University of Toronto, Toronto, Canada; 2 School of Public Health and Health Systems, University of Waterloo, 200 University Ave W, Waterloo, ON Canada; 3 Public Health Ontario, Toronto, Canada; 4 Dalla Lana School of Public Health, University of Toronto, Toronto, Canada

**Keywords:** Relative validity, Sugary drinks, Sugar sweetened beverages, Dietary assessment, Food frequency screener

## Abstract

**Background:**

The contribution of beverages to overall diet is of increasing interest to researchers and policymakers, particularly in terms of consumption of drinks high in added sugars; however, few tools to assess beverage intake have been developed and evaluated. This study aimed to evaluate the relative validity of a new online Beverage Frequency Questionnaire (BFQ) among young adults in Canada.

**Methods:**

A cross-sectional relative validation study was conducted among young adults aged 16–30 years (*n* = 50). Participants completed a 17-item BFQ, a 7-day food record (7dFR), and a single-item measure of sugary drink intake. Pearson correlations and paired t-tests were used to evaluate correlation and agreement between the measures for 17 individual beverage categories, total drink consumption, total alcoholic beverage consumption, and two definitions of drinks with excess sugars. Cognitive interviews were conducted to examine participant interpretation and comprehensiveness of the BFQ.

**Results:**

Estimates of beverage intake based on the BFQ and the 7dFR were highly correlated, specifically for the total number and volume of beverages consumed, total alcoholic beverage consumption, sugary drink intake, and each of the 17 beverage categories with 3 exceptions: coffee or tea with sugar or cream, specialty coffees, and hard alcohol with caloric mix. Paired t-tests between the BFQ and the 7dFR indicated that the average reported volume was significantly different only for sweetened fruit drinks. The single-item measure of sugary beverage intake was not significantly correlated with the 7dFR. Cognitive interviewing demonstrated high comprehension levels, and confirmed the appropriateness of the BFQ beverage categories and sizes.

**Conclusions:**

Overall, the results suggest that the BFQ performed well relative to a 7dFR and had high usability among this study population, indicating its promise for collecting population-level data on beverage intake, including sugar-sweetened beverages, which are known indicators of diet and health.

**Electronic supplementary material:**

The online version of this article (10.1186/s12937-018-0380-8) contains supplementary material, which is available to authorized users.

## Background

Beverages, especially those high in free sugar, are of particular and growing interest among researchers and policymakers. A well-established body of evidence has demonstrated the link between high consumption of sugar-sweetened beverages (SSBs), which are beverages containing added sugars, and diseases such as type 2 diabetes, metabolic syndrome, cardiovascular disease, dental caries, and several cancers [1–11]. Sugar-sweetened beverages have been associated with these diseases primarily through their contribution to excess weight gain, [12, 13] and contribute substantial caloric energy, have low nutrient density, are associated with poorer quality diets, and offer low satiety in comparison to foods [14, 15]. Additionally, there is growing evidence suggesting that 100% juice, which contains free sugars, has similar impact as SSBs in terms of dietary compensation and the effects of sugars in juice on diabetes and other health conditions [5, 13, 16, 17].

Sugary drinks, which include SSBs and 100% juice, are the single leading source of sugar in Canadians’ diets. In 2004, Canadians reported consuming an average of 371 g of sugary drinks per day [18–20]. Canadian youth (14 to 18 years) and young adults (19 to 30 years) were the largest consumers of sugary drinks, consuming an average of 643 mL and 500 mL per day, respectively. Regular carbonated soft drinks have long dominated the Canadian sugary beverage market. However, an increasing number of novel beverage categories are being introduced, which have partially offset declines in sales of traditional sugary drinks, such as carbonated soft drinks, fruit drinks and 100% fruit juice [21]. Between 2004 and 2015, sales of flavoured water, flavoured milk, drinkable yogurt, and energy drinks grew from negligible proportions to accounting for approximately 12% of all sugary drink sales volume in Canada [21].

Given the unique contribution of sugary drinks to key metabolic diseases, and their prominent role in Canadians’ diets, it is important to accurately monitor beverage consumption at a population level. Beverage intake has historically been measured using a variety of tools. Both 24-h recalls and dietary records (or ‘food diaries’) provide comprehensive data on foods and beverages consumed by participants in the previous day or over a specified period of time, and have been used to derive beverage intake data [22, 23]. However, such tools can be resource intensive to administer, and place a high burden on participants’ time and effort. Online versions of 24-h recalls and records, such as the Automated Self-Administered 24-h Dietary Assessment Tool (ASA24), have been developed to reduce the cost and enhance the feasibility of collecting such comprehensive dietary intake data, from which beverage intake information can be obtained [24]; however, the time required to complete such tools may be prohibitive for some studies. To further enhance the feasibility of collecting beverage intake data from large samples, smaller multi- or single-item beverage frequency questionnaires have been developed to examine changes in beverage intake over time [25, 26].

Frequency-based screeners can play an important role in population-based research. Although frequency-based measures have the limitation of requiring respondents to ‘average’ intake, which imposes cognitive challenges and contributes bias, depending on the number of items included, they can be considerably shorter and thus may be more feasible than 24-h recalls to administer in some situations. Further, screeners can be used to assess ‘typical patterns’ of intake, which are of interest in most population-level research [27–29]. However, the existing beverage frequency measures have several important limitations. For example, tools may include general beverage categories without breaking out sub-categories of interest, such as sugary drinks. Existing tools may also have inadequate coverage of new or emerging beverage categories, such as vitamin waters or non-caloric drinks [30–32]. Additionally, most beverage frequency tools use paper-based methods and have not been adapted or tested for online administration. A large majority have been evaluated only in US populations, using US-based beverage container sizes and measurements. Most existing beverage frequency tools do not utilise visual cues for indication of beverage sizes, instead relying on participants to recall numeric sizes, such as “355 mL”, or do not inquire about container or portion size [30–32]. In addition, beverage measures have historically failed to include alcoholic beverages, which are a major contributor to intake of both energy and sugars, particularly among those more likely to engage in binge drinking behaviours, such as adolescent and young adult populations [33–36]. Finally, it is challenging to accurately capture intake of episodically-consumed beverages, such as alcohol and energy drinks, as well as sugary drinks, among occasional consumers using 24-h recalls or food records collected for one or a few days.

Given the increasing political and public health focus on sugary drinks, it is important that effective and efficient measures are available with which to characterize beverage consumption at a population level. The objectives of the current study were to compare a newly developed Beverage Frequency Questionnaire (BFQ) to a 7-day dietary record, to examine the ability of the tool to capture the frequency of consumption of drinks with added and free sugar, and to examine whether the BFQ performed better than a single-item measure that has been used in population-level surveys to assess intake of SSBs. It was hypothesized that there would be good agreement between the BFQ and the 7-day food record, and that the BFQ would provide more accurate estimates of beverage intake relative to the 7-day record than the single-item measure that has been widely used in population-based surveys to date.

## Methods

### Participants

This study was conducted in April and May 2016 with a convenience sample of 50 young adults from a university campus and its surrounding community in southwestern Ontario, Canada. Participants were eligible if they were between 16 and 30 years old, and could read and speak English. Quotas were set to ensure an even proportion of males and females. Participants were recruited until the target sample size was reached, and were remunerated with $40 CAD. This study received ethics clearance through a University of Waterloo Research Ethics Committee (#21304).

### Study design

Eligible participants attended two group sessions. During Visit 1, participants provided written consent and completed a brief socio-demographic survey and the BFQ. At this session, participants were provided with verbal and written instructions for completing a food record, and were asked to complete the food record for the next 7 days. Participants returned for Visit 2 eight days after the initial visit to submit their food record, complete a single-item measure of SSB intake and a second BFQ, and participate in cognitive interviewing. All surveys were self-administered using a provided laptop with the pre-loaded online tools.

### Measures

#### Beverage Frequency Questionnaire (BFQ)

The BFQ is an online beverage frequency screener that examines consumption of 17 categories of beverages, including alcoholic beverages (see Additional file 1: Figure S1 for the full list of categories and examples provided in the BFQ). Categories were modelled after previous existing questionnaires for beverage consumption, [30–32] with substantial adaptations to ensure that categories could adequately discern between sweetened and unsweetened beverages, caloric and non-caloric beverages, and individual beverage categories. Within caloric and non-caloric drinks, distinctions were made to distinguish between key beverage attributes (e.g., carbonated vs. non-carbonated, heavily caffeinated products).

**Fig. 1:**
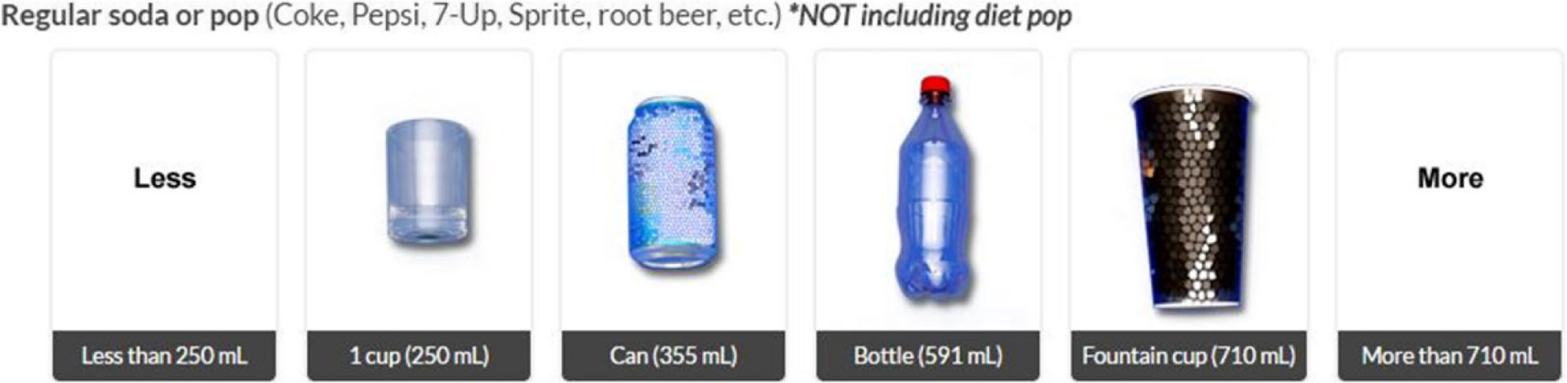
Example of images used in the BFQ

First, the survey asked participants, “During the PAST 7 DAYS, how many times did you drink each of the following beverages?” for each drink category. Next, for each of the categories that the participant consumed, the participant was asked, “On average, how much did you usually drink each time?” and was shown a series of images, adapted from ASA24, of commonly-used containers with the volume in millilitres below the container [24]. Response options for ‘Less’ than the smallest image amount and ‘More’ than the largest image amount were also available. Figure 1 provides an example of container sizes for the “regular soda or pop” beverage category. Additional information regarding the online format is available from the corresponding author.

#### Seven-day food record *(*7dFR)

Participants were provided with a standard food record booklet with spaces for a description of the specific food or beverage item consumed (including brand information, when possible), the location at which it was prepared, and the number and portion size or amount that was consumed. Participants completed the food record for 7 days, beginning the day after Visit 1. Participants were instructed to record all meals, beverages and snacks consumed each day, ideally in real-time as foods and beverages were consumed, and were asked to provide as much detail as possible.

#### Single-item measure of sugar-sweetened beverage intake

Prior to completing the BFQ at Visit 2, participants were asked, “During the PAST 7 DAYS, how many drinks with added sugar did you have, such as pop, fruit drinks, sports drinks, vitamin waters, energy drinks and specialty coffees? Do NOT count diet or sugar-free drinks. Do NOT include today.” This measure did not specifically include 100% fruit juice, and was meant to capture SSBs.

#### Socio-demographic questionnaire

Participants were queried about sex, age, education level, and race/ethnicity, as well as self-reported weight and height, which were used to calculate body mass index (BMI), according to World Health Organization (WHO) categories of underweight, normal weight, overweight and obese [37].

#### Cognitive interviewing

Cognitive interviewing was conducted with participants to assess the comprehensiveness of the BFQ, their comprehension of the BFQ and interpretation of the instructions, and to examine how participants used the tool. After completing the BFQ at Visit 2, participants were provided with paper copies of the screens they had seen on the computer when completing the tools. Cognitive interviewing was conducted in small groups of one to four participants by a trained research assistant, who took notes on responses.

First, participants were asked if they had trouble recalling the number of drinks for any specific categories, if there were any drinks not on the current list that they felt should be added, and whether or not they were thinking about each of the 7 days individually or gave an estimated amount based on their usual patterns. Participants were also asked whether they recalled seeing images for serving sizes, and, if so, whether they used the shape of the container or the amount written under the container, or both, to identify their serving size. Lastly, they were asked if, over the course of the week, they had the same type of drink in different sizes of containers (e.g., a can of pop one day and a bottle the next day), and if so, how they chose which container/size to select.

### Analysis

#### Food records

Data from the 7dFRs were coded by a trained research assistant to identify incidences of consumption and amounts consumed of beverages in the categories included in the BFQ, as well as other beverages not captured by the BFQ. Because the BFQ relates to a 7-day period, the volume consumed within each beverage category was summed over the 7-day period. When beverages were consumed in dining establishments outside of the home, restaurant websites were used to estimate the volume of beverage containers (e.g., for fountain drinks). When the frequency of consumption was not reported, it was assumed that the beverage category was consumed zero times. When size was not reported in the food record, the smallest size for the specific beverage category in the BFQ was used. Assumptions were required in at least one instance in 14 food records (4 instances for the frequency of consumption and 14 instances for the volume consumed).

#### Beverage Frequency Questionnaire (BFQ)

Data from the BFQ estimated of the number of times each drink category was consumed. This number was multiplied by the usual serving size selected (in mL) to estimate the total volume consumed over the past 7 days for each beverage category. When a participant reported consuming less than the smallest size provided, the amount was estimated as 50% of the smallest size option in that beverage category; when a participant reported consuming more than the largest size provided, the amount was estimated as 125% of the largest size. For example, if a participant reported consuming less than the smallest size of regular soda or pop (250 ml), it was assumed that they consumed 125 ml of regular pop or soda; if they reported consuming more than the largest size of regular soda or pop (710 ml), it was assumed they consumed 888 ml of regular soda or pop. Some questions were combined to create definitions for beverage categories, such as alcoholic drinks, which included: beer, cider or coolers; wine (red or white); hard alcohol with mix, cocktails that have calories; and hard alcohol with no mix or non-caloric mix.

#### Definitions of beverages with excess free and added sugars

Two definitions were used to classify beverages with excess sugar content. The first was a typical definition of *SSBs*, which included: regular soda or pop; sweetened fruit drinks; flavoured waters or vitamin waters with calories; sports drinks; energy drinks; and, specialty coffees. A more comprehensive definition of *sugary drinks* was also utilized, which included the aforementioned beverages, as well as: 100% fruit or vegetable juice; chocolate milk; regular coffee with cream or sugar; and hard alcohol with mix or cocktails that had calories. Analyses were conducted to examine the correlation between the food record and the BFQ for the two definitions of beverages with excess sugar content.

#### Statistical analysis

Pearson correlations were used to examine the relationship between the number of times (drinks) and total volume of beverages consumed per 7-day period, as reported in the food record and the BFQ for each drink category, for the *SSB* and *sugary drinks* definitions, for alcoholic drinks, and for all drinks combined. Analyses comparing the *SSB* and *sugary drinks* definitions were also conducted stratified by race/ethnicity. Agreement and correlations were also examined comparing the single-item measure to the 7dFR using paired samples t-tests and Pearson correlations. Cognitive interviewing notes were summarized by the interviewer, and suggestions for amendments to the BFQ were collated. Bland-Altman plots were generated to compare the measurements from the two tools.

## Results

### Participant characteristics

The final sample of 50 participants were primarily normal weight university students, with diverse racial/ethnic backgrounds (Table 1).

**Table 1:** Sample characteristics

		(Frequency)
Mean Age (SD)	22 (2.99)	(50)
Gender		
Male	50.0%	(25)
Female	50.0%	(25)
Body Mass Index (BMI) category		
Underweight	6.0%	(3)
Normal weight	72.0%	(36)
Overweight	12.0%	(6)
Obese	8.0%	(4)
Not stated	2.0%	(1)
Race/ethnicity		
White	42.0%	(21)
Other	58.0%	(29)
Educational attainment		
High school or less	22.0%	(11)
Some university, no degree	42.0%	(21)
Completed university degree	30.0%	(15)
Postgraduate degree	6.0%	(3)

### Beverage consumption

The number of BFQ beverage categories reported by participants ranged from 0 to 7, out of a possible 17 categories, with an average of 3.16 categories (s.d. = 1.74). Three participants reported not consuming any BFQ beverages (note: plain water was not included as a category).

When the data from the BFQ and 7dFR were compared (Table 2), both the number of drinks and the volume of drinks were positively correlated between the BFQ and 7dFR for all 17 beverage categories, with three exceptions: coffee or tea with sugar or cream, specialty coffees, and hard alcohol with caloric mix. There were also positive correlations between the number and volume of drinks in the BFQ and food record for all drinks combined and all alcoholic drinks combined. Paired t-tests between the BFQ and the 7dFR indicated that the average volume was not significantly different for any category except sweetened fruit drinks. When qualitatively compared, food records from 16 participants included beverages that were not captured in the BFQ: 11 participants reported consuming smoothies and five reported consuming protein drinks, neither of which had dedicated categories on the BFQ.

**Table 2:** Average number of beverages and volume consumed, according to the Beverage Frequency Questionnaire (BFQ) and 7-day food record (7dFR)

Beverage category	Mean # of drinks (s. e.)		r_p_ # of drinks	*p*-value	Mean total volume in ml (s. e.)		Mean volume difference between BFQ and Record (s. e.)	r_p_ total volume	*p*-value	t-test for total volume	*p*-value
	7dFR	BFQ			7dFR	BFQ					
1. Regular soda or pop	0.78 (0.27)	0.86 (0.26)	0.58	< 0.001	277 (99)	388 (133)	+ 298 (112)	0.51	< 0.001	−0.93	0.36
2. Diet soda or pop	0.00	0.06 (0.0)	–	< 0.001	0 (0)	21 (21)	21 (21)	–		−1.00	0.32
3. 100% fruit/vegetable juice	2.00 (0.46)	1.88 (0.45)	0.56	< 0.001	506 (117)	494 (115)	387 (104)	0.48	< 0.001	0.10	0.92
4. Fruit drinks with added sugars	1.06 (0.34)	0.52 (0.18)	0.46	< 0.001	359 (94)	154 (57)	295 (72)	0.56	< 0.001	2.62	0.01
5. Flavoured water/vitamin water with calories	0.08 (0.48)	0.06 (0.04)	0.91	< 0.001	37 (23)	35 (26)	13 (8)	0.95	< 0.001	0.20	0.84
6. Sports drinks	0.12 (0.06)	0.22 (0.10)	0.60	< 0.001	82 (46)	124 (56)	63 (31)	0.82	< 0.001	−1.33	0.19
7. Energy drinks	0.12 (0.07)	0.12 (0.06)	0.91	< 0.001	51 (29)	57 (29)	23 (13)	0.89		−0.40	0.69
8. Calorie-free drinks	0.00	0.02 (0.02)	–	< 0.001	0 (0)	12 (12)	12 (12)	–		−1.00	0.32
9. Unsweetened milk or alternatives	1.60 (0.45)	2.08 (0.56)	0.47	< 0.001	478 (135)	640 (233)	469 (178)	0.59	< 0.001	−0.86	0.39
10. Chocolate or sweetened milk and alternatives	0.90 (0.27)	0.88 (0.37)	0.58	< 0.001	336 (109)	255 (112)	311 (103)	0.49	< 0.001	0.73	0.47
11. Coffee/tea with cream or sugar	2.32 (0.56)	2.06 (0.39)	0.20	0.1	833 (223)	517 (135)	747 (225)	0.13	0.37	1.29	0.20
12. Coffee/tea no cream or sugar	1.66 (0.48)	1.74 (0.61)	0.65	< 0.001	570 (197)	729 (282)	588 (165)	0.76	< 0.001	−0.87	0.39
13. Specialty coffee	0.54 (0.16)	0.36 (0.14)	0.23	0.11	211 (64)	116 (43)	208 (62)	0.26	0.07	1.41	0.16
14. Beer/cider/coolers	0.58 (0.21)	0.60 (0.20)	0.92	< 0.001	271 (106)	250 (78)	143 (50)	0.87	< 0.001	0.40	0.69
15. Wine	0.10 (0.06)	0.12 (0.07)	0.60	< 0.001	58 (42)	29 (18)	57 (36)	0.50	< 0.001	0.82	0.42
16. Hard alcohol with caloric mix	0.16 (0.09)	0.28 (0.13)	0.18	< 0.001	56 (33)	70 (31)	40 (21)	0.78	< 0.001	−0.64	0.53
17. Hard alcohol no mix or non-caloric mix	0.30 (0.19)	0.24 (0.14)	0.18	0.23	9 (11)	13 (8)	24 (12)	0.16	0.25	0.51	0.61
Sugar-sweetened beverages^a^	2.70 (0.51)	2.14 (0.40)	0.53	< 0.001	1017 (183)	875 (173)	634 (147)	0.54	< 0.001	0.82	0.41
Sugary drinks^b^	8.08 (1.18)	7.24 (0.94)	0.63	< 0.001	2749 (392)	2211 (316)	1368 (291)	0.55	< 0.001	1.57	0.12
All alcoholic drinks	1.14 (0.31)	1.24 (0.34)	0.58	< 0.001	405 (120)	361 (96)	44 (76)	0.78	< 0.001	0.58	0.57
All drinks	12.32 (1.40)	12.10 (1.20)	0.62	< 0.001	4145 (484)	3904 (463)	241 (428)	0.59	< 0.001	0.56	0.57

^**a**^Includes: regular soda or pop; sweetened fruit drinks; flavoured waters or vitamin waters with calories; sports drinks; energy drinks; and specialty coffees

^b^Includes: regular soda or pop; sweetened fruit drinks; flavoured waters or vitamin waters with calories; sports drinks; energy drinks; specialty coffees; 100% fruit or vegetable juice; chocolate milk; regular coffee with cream or sugar; and hard alcohol with mix or cocktails that had calorie

### Definitions of drinks with excess sugar content

**Fig. 2:**
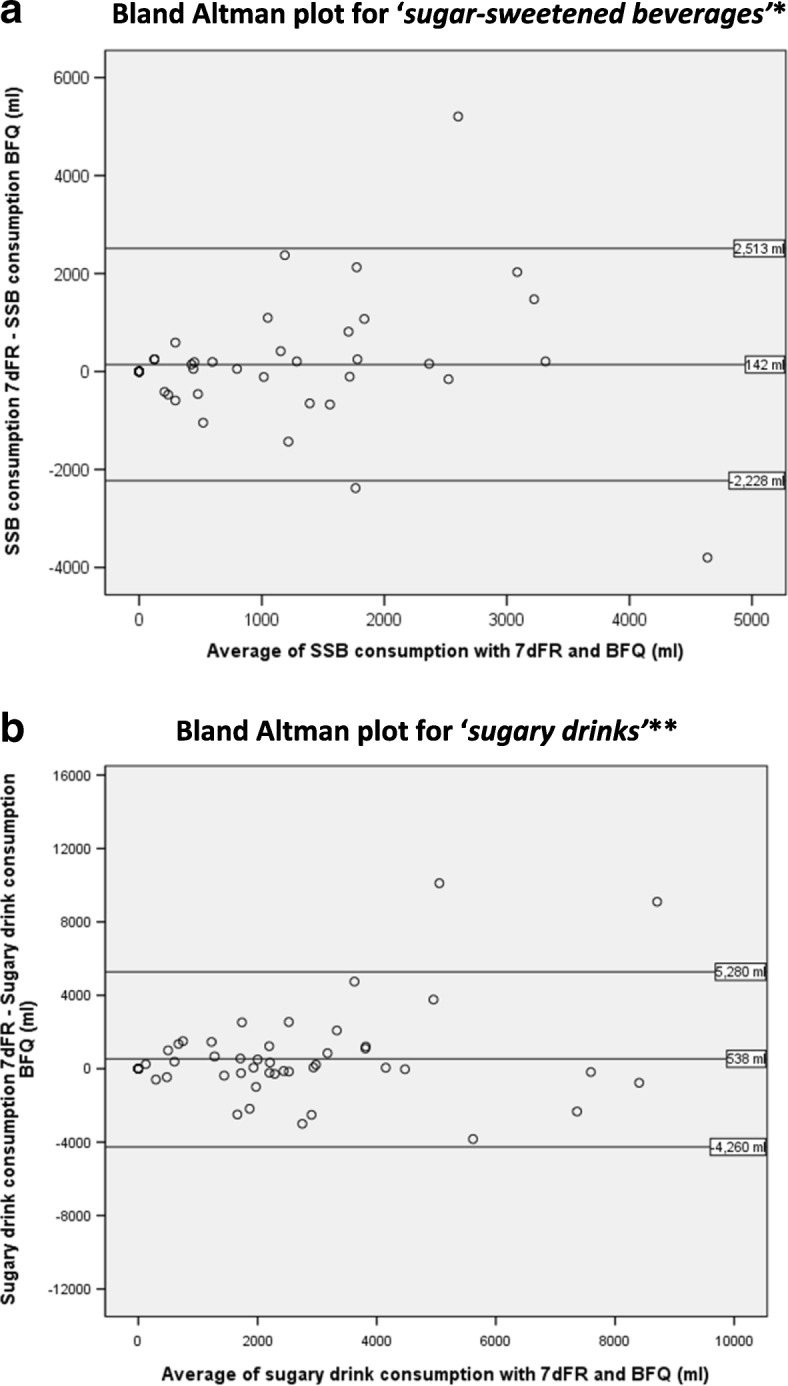
**a** Bland Altman plot for ‘sugar-sweetened beverages’*. **b** Bland Altman plot for ‘sugary drinks’**

Correlations between the BFQ and 7dFR for the total number of beverages and the total volume consumed were significant for both *sugar-sweetened beverages* (regular soda or pop; sweetened fruit drinks; flavoured waters or vitamin waters with calories; sports drinks; energy drinks; and specialty coffees) and *sugary drinks* (sugar-sweetened beverages plus 100% fruit or vegetable juice; chocolate milk; regular coffee with cream or sugar; and hard alcohol with mix or cocktails that had calories). When stratified by race/ethnicity, trends were similar and all findings remained significant within each category (results not shown). Bland-Altman plots suggested no proportional bias with the limited definition of SSBs (*t* = 0.32, *p* = 0.75) and for the sugary drinks definition (*t* = 1.78, *p* = 0.08). Figure 2 shows that 94% of the differences for both SSBs and sugary drinks, respectively, fell within the limits of agreement (2 standard deviations from the mean).

### Comparison to single-item measure

The mean number of *SSBs* reported over 7 days was 3.66 (s.d. 4.39; range: 0–18) based on the single-item measure, compared to 2.70 (s.d. 3.59; range: 0–14) based on the 7dFR, and 2.14 (s.d. 2.85, range: 0–15) based on the BFQ. While there was no significant difference in the mean number of SSBs consumed based on the single-item measure and the 7dFR (*t* = 1.36, *p* = 0.18), there was not a significant correlation between the number of SSBs based on the single-item measure and the 7dFR (0.23, *p* = 0.10).

### Cognitive interviewing

Overall, 32% of the participants (*n* = 16) reported that they had trouble reporting the number of drinks for the categories provided in the BFQ. When asked how they recalled the number of drinks they consumed in the last week, 54% (*n* = 27) of the participants reported that they considered each of the past 7 days, 22% (*n* = 11) gave an estimate based on their usual patterns, and 24% (*n* = 12) reported using a combination of considering each of the past 7 days and estimating based on their usual patterns. In addition, 20% (*n* = 10) of participants reported that they could not find a drink on the list and felt that it should be added to the BFQ (*n* = 3 suggested protein drinks and n = 3 suggested smoothies).

In total, 22% of the 49 participants who viewed images depicting typical container sizes for at least one beverage category had trouble selecting an image for the beverage category. Participants reported that the container or volume of beverages they consumed was not captured in the images and they had to guess the closest size. When participants were asked whether they tried to match the shape of the container or the amount written under the container when they selected an image, 27% indicated they tried to match the shape, 29% tried to match the amount, and 45% used both the shape and the amount written under it. Overall, 2 participants (4%) reported that alcohol was particularly hard to recall due to oddly shaped glasses that they were served in.

Across the sample, 42% (*n* = 21) reported that they consumed the same type of beverage in different sizes of containers over the course of the week. When selecting an image, 36% (*n* = 18) of participants reported that they selected the size that they consumed the most often, and 3 selected the larger container size.

## Discussion

The results indicate strong correlation and agreement between the BFQ and the 7dFR for the vast majority of beverage categories and for the overall definitions of *sugar-sweetened beverages* and *sugary drinks*. These results are very similar to a 2010 validation study of a paper-based beverage frequency questionnaire that also did not find significant correlations for hard alcohol, or coffee with cream or sugar when compared to a 4-day food record; however, the current study found higher correlations across all beverage categories [31]. Coffee with cream or sugar was among the most frequently consumed beverages, and was consumed in large volumes, which may contribute to poorer recall of instances of consumption and greater likelihood of having to select an average container size that may not precisely reflect all of the various container sizes consumed. Given that the overall contribution of added sugar or calories from this category are likely to be small, this is unlikely to influence estimates of energy or added sugar intake. The reasons for poor recollection of consumption of specialty coffees and caloric alcoholic drinks is unclear, although both were consumed relatively infrequently. The cognitive interview data suggest that this could be due to alcohol being served in unusual beverage containers, which may create challenges in completing a frequency-type questionnaire. Future iterations of the BFQ may consider additional container sizes and shapes for alcoholic beverages and additional prompts to improve recall of coffee and tea with cream or sugar, and coffee and tea with cream or sugar will be removed from the definition of *sugary drinks*.

The BFQ performed better relative to the 7dFR than the single-item measure in assessing SSB and sugary drink intake. While there are advantages to using an abbreviated measure, such as the single-item measure, in terms of time and respondent burden, the findings indicate a more comprehensive tool, such as the BFQ, can improve recall and estimation of the frequency and amount of beverages consumed, without creating the level of participant burden that might be associated with administering 24-h recalls or food records to capture the total diet. However, the BFQ obviously does not provide information on other aspects of the diet that may be of interest, for example, for considering how beverage intake patterns relate to other characteristics of eating patterns, which would be captured in a 24-h recall or food record.

Most participants did not report difficulty using the BFQ, and used the BFQ as intended by considering each of the previous 7 days when estimating intake, rather than basing estimates solely on usual patterns. Additionally, the participants typically selected the size consumed most often, rather than the largest or smallest size consumed, which should decrease any bias towards over- or under-reporting. The cognitive interviewing indicated that participants benefited from having the images to guide estimation of container size, as more than half of participants used both the container shape and the volume amount to guide their selection; however, this study did not explicitly test whether or not this improved volume estimations, and portion size estimation is known to be a challenging aspect of dietary assessment.

With regards to face validity, the results suggest that the BFQ reasonably captures most beverages that are consumed by this age group, with the exception of protein shakes and smoothies. During the cognitive interviewing, participants noted that these two drink categories were missing from the beverage questionnaire. These beverages’ potentially high sugar contents and their reported frequent consumption position them as potentially meaningful sources of dietary sugar. A revised version of the BFQ for use in future research includes these categories. Few young adults in this sample consumed calorie-free drinks, consistent with national data based on dietary recalls indicating highest consumption among adults over 30 years of age [19, 20]. Consumption of these types of beverages may become more prevalent as efforts to reduce dietary sugar intake increase with age.

### Study limitations and strengths

This study assessed relative validity of the BFQ by comparing intake estimates to another self-report measure. 7dFRs are one of the most widely used methods for capturing self-reported dietary intake, but may be subject to reactivity in addition to other sources of bias [38]. The correlations between the BFQ and 7dFR data may reflect agreement both in terms of true intake as well as biases that may be common to data collected using the two tools (e.g., underreporting of certain categories of beverages due to social desirability biases). An objective measure is needed to overcome these challenges; however, there is no biomarker for beverage intake and observation is not feasible for the 7-day period of study in this research, and can also lead to reactivity. Future research may make use of biomarkers of sugar intake to shed further light on the ability of tools such as the BFQ to capture intake of total, free, and added sugars [39, 40].

Further, the order of administration of tools may have affected responses. For example, the completion of the 7dFR may have led to increased accuracy on the BFQ completed at the 2nd visit, possibly inflating the correlations between the BFQ and the food record. Additionally, the sample size was relatively small and a small number of respondents reported consumption of beverages in some categories, lending uncertainty to the estimates for those categories. The relatively demanding task of recording dietary intake over 7 days may have resulted in sample selection bias in that those with knowledge and interest in nutrition were motivated to participate; however, this was potentially reduced by the remuneration provided as an incentive to participate. While the population under study was restricted primarily to university students, the study population included a large sample of non-White participants, and included those with high variation in beverage intake behaviours, increasing the generalizability to the young adult population in Canada.

### Tool limitations and strengths

Given the challenge of recalling plain water consumption, this was not included in the BFQ; therefore, the BFQ cannot capture shifts from caloric beverages to plain water. Further, the BFQ does not provide data on the total diet, limiting its use for examining beverage intake in relation to other characteristics of eating patterns. Lastly, the tool was not developed to examine other characteristics of beverages, such as caffeine consumption or added components such as vitamins and minerals, which may be of interest to some researchers. Strengths of the BFQ include the inclusion of specific categories of beverages with attention to contributions to intake of energy and sugars, as well as categories frequently consumed in the current marketplace. The tool captures consumption of beverages that may be episodically consumed, such as energy drinks. Finally, this tool captures both alcoholic and non-alcoholic drinks. Given the high amounts of sugar in some alcoholic drinks and the contribution of alcohol to calorie intake, this is an important addition to the literature. Additionally, the revised version of the BFQ has been translated into French and Spanish (although it has only been evaluated in English to date), and has been adapted for use in other countries (United States, Mexico, United Kingdom and Australia) by changing brand names and portion/container sizes. Future work with the tool will include developing country-specific category averages of total sugar, free/added sugar, and calories, using branded food and beverage databases that can be applied to beverage categories to estimate added sugar and energy consumption from beverages.

## Conclusions

Overall, this study showed that the online BFQ performs well relative to a more comprehensive measure of intake for capturing intake of regularly-consumed beverage categories, including the frequency and volume of beverages with excess sugars, and is a promising tool to measure population-level beverage intake. The unique features of this tool, including its online application, the use of images to improve reporting of serving sizes, and the reference period of 7 days to capture episodically-consumed beverages, make this a novel tool for researchers and practitioners seeking to gather data about beverage consumption patterns.

## Additional file

Additional file 1: **Figure S1.** Is available from the “Online Supporting Material” link in the online posting of the article and from the same link in the online table of contents at jn.nutrition.org. (DOCX 113 kb)
