# Supplementary material for: Evaluation of the online Beverage Frequency Questionnaire (BFQ)
Source: Nutr J. 2018 Aug 1;17:73. doi: 10.1186/s12937-018-0380-8 (PMC6090967; doi:10.1186/s12937-018-0380-8)

**Supplemental Figure S1. Beverage Frequency Questionnaire beverage categories and examples provided.**


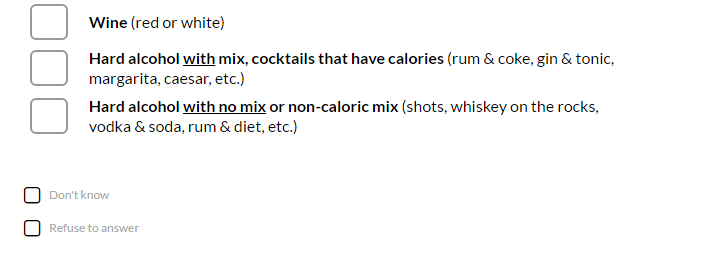

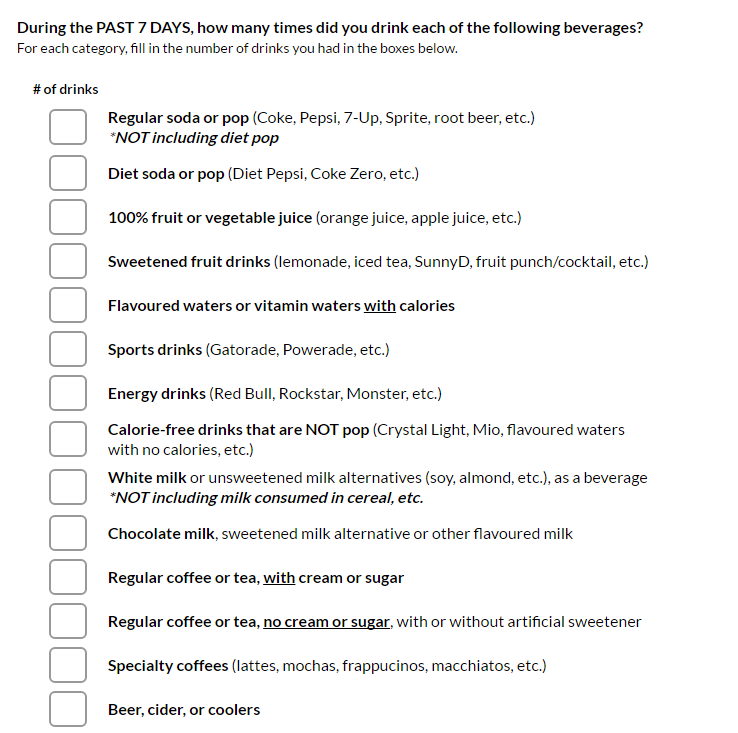

Supplement: Supplementary file 1 — Figure S1. Is available from the “Online Supporting Material” link in the online posting of the article and from the same link in the online table of contents at jn.nutrition.org. (DOCX 113 kb) [file 12937_2018_380_MOESM1_ESM.docx]
